# MRgFUS thalamotomy for the treatment of tremor: evaluation of learning curve and operator’s experience impact on the procedural and clinical outcome

**DOI:** 10.1007/s00701-023-05510-z

**Published:** 2023-02-10

**Authors:** F. Bruno, E. Tommasino, L. Pertici, V. Pagliei, A. Gagliardi, A. Catalucci, F. Arrigoni, P. Palumbo, P. Sucapane, F. Pistoia, C. Marini, A. Ricci, A. Barile, E. Di Cesare, A. Splendiani, C. Masciocchi

**Affiliations:** 1 Department of Biotechnological and Applied Clinical Science, University of L’Aquila AQ, L’Aquila, Italy; 2 Neurology, San Salvatore Hospital, L’Aquila, Italy; 3 Department of Clinical Medicine, Public Health, Life Sciences and Environment Life, University of L’Aquila, L’Aquila, Italy; 4 Neurosurgery, San Salvatore Hospital, L’Aquila, Italy

**Keywords:** Tremor, Essential tremor, Parkinson’s disease, Vim thalamotomy, MRgFUS

## Abstract

**Background:**

MRgFUS Vim ablation is increasingly used for the treatment of tremor in ET e PD patients but there is little published research on the importance of operator experience in this procedure. This study aims to evaluate the learning curve and the influence of the operator experience on the procedural and clinical outcomes.

**Methods:**

We retrospectively evaluated 90 patients (38 ET, 52 PD) submitted to MRgFUS unilateral thalamotomy in the period between February 2018 and July 2020. Clinical endpoints, procedural times, and technical parameters were recorded in all procedures. Based on the time of treatment, patients were divided into three groups of 30 units each, comparing all variables between each time period group.

**Results:**

In Group A, the average patient preparation time was 120.6 min, the treatment time was 105.2 min, the number of was sonications 14.1, and the mean target shifts 3.1. In Group B, the mean preparation time was 105.5 min, the treatment time was 89.5 min, the number of sonications was 13.2, and the target shifts 3.0. Group C showed inferior values of preparation time (101.9 min), treatment time (71.7 min), numbers of sonications (10.6), and shifts (1.7). Thalamotomy-related complications occurred in 9 patients of Group A, 2 of Group B, and 5 of Group C. Tremor relapse occurred in 7 patients of Group A, 3 of Group B, and 2 of Group C. The days of hospitalization were comparable in the three groups.

**Conclusions:**

The operators experience is associated with the improvement of clinical and procedural outcome in MRgFUS thalatomy for the treatment of ET and PD tremor.

## Introduction

Since the recent FDA approval after several preclinical and clinical trials, Magnetic Resonance guided Focused Ultrasound Surgery (MRgFUS) Vim thalamotomy is rapidly becoming established as an effective treatment option for tremor in patients with essential tremor (ET) and tremorigenic Parkinson’s disease (PD) [1, 22].

Procedurally, MRgFUS thalamotomy is a minimally invasive ablation technique, derived from stereotactic surgical techniques, that involves the use of high-intensity focused ultrasound delivered through the intact skull to specific thalamic nuclei [6, 18–20]. FUS has long been used in interventional radiology primarily for ablative therapy of tumours in oncology [3–5, 15, 16]. The use in the field of neuroscience has made this treatment increasingly transversal between different professional figures (neuroradiologist, neurologist, neurosurgeon); if on the one hand, clinical skills are necessary for the intraprocedural assessment of the patient, on the other hand, it is essential to know how to master the imaging and technical setting of ultrasounds. It is also important to have a cultural background of stereotactic brain surgery. In clinical practice, treatment involves a disciplinary team, where the primary operator is usually a neuroradiologist [2, 6, 12, 13, 21].

In recent years, surgical techniques in all areas of the body have evolved and changed with the advent of robotics technology and imaging guidance. It was therefore necessary to build a scientific literature on the impact of these new approaches on procedural results and on the operator’s learning curve [10, 14].

To the best of our knowledge, no previous studies examined this impact on MRgFUS Vim thalamotomy; in this paper, we therefore tried to highlight the importance of the operator experience, critically examining the learning curve and its effect on the procedural and clinical outcomes in patients with tremor.

## Methods

### Study population

We retrospectively analyzed all patients undergoing MRgFUS Vim unilateral ablation for the treatment of disabling tremor from February 2018 to July 2020. The study population consisted of 90 patients (68 M, 22F), with a median age of 70 years (95% CI 67.8–72, range 36–90) and median disease duration of 15.5 (95%CI 11–19.67, range 2–47). Demographic data are reported in Table 1.

**Table 1:** Baseline characteristics

Groups	A	B	C	*P*-value
Sex	9F-21 M	7F-23 M	6F-24 M	0.55 A–B 0.37 A–C 0.75 B–C
Pathology	15ET 15PD	12ET 18PD	11ET 19PD	0.43 A–B 0.29 A–C 0.79 B–C
Mean age (years)	66.5	71.0	67.1	0.2 A–B 0.9 A–C 0.2 B–C

All patients presented with disabling tremor, refractory to drug therapy (38 patients with essential tremor (ET), 52 patients with tremorgenic Parkinson’s (PD) and were clinically selected by a neurologist expert in movement disorders (PS, FP). Sixty patients underwent left thalamotomy (right hand tremor), and 30 patients right thalamotomy (left hand tremor). Based on the time of treatment, patients were divided into three groups of 30 units each: the first group included patients enrolled from February 2018 to January 2019 (Group A), the second group from January 2019 to September 2019 (Group B), the third from September 2019 to July 2020 (Group C).

From clinical and procedural reports, clinical and procedural data and parameters were retrieved and evaluated in all patients.

### Clinical parameters

The parameters evaluated before treatment and at follow-ups were demographic data (age, sex, pathology), the Fahn-Tolosa-Marin scale for tremor (FTM), the skull density ratio (SDR) between cortical bone and cancellous bone (values > 0.3 were considered suitable for treatment), days of hospitalization, and thalamotomy-related complications (paresthesia, postural instability, and transient hemiparesis, ataxia, dysarthria, motor, and facial deficits, painful dystonia and strength deficit on the treated side). We evaluated the part A of the FTM, rating resting, postural and action tremor of the arm treated/to be treated (0–12 points); tremor relapses were extrapolated by comparing the last value of the Fahn-Tolosa-Marin scale with the follow-up values at 24 h, considering relapse as an increase of more than 5 points.

### Procedural parameters

All treatments were performed at our Institution with an ExAblate Neuro machinery (NeuroAblate 4000, InSightec Ltd, Carmel-Tirat, Israel) according to the treatment procedures described elsewhere, by the same team of interventional radiologists (FB, AC, FA) [1].

### Patient preparation time

The patient preparation includes trichotomy, premedication, and helmet positioning. On the day of the procedure, a complete scalp trichotomy is performed to eliminate any type of ultrasound diffraction of the skin. The premedication of the patient is carried out with intravenous administration of paracetamol, cortisone, and ondansetron. After the injection at the level of the periosteum of a mixture of anesthetics, the stereotactic frame (CRW Integra) is positioned using screws inserted anteriorly at the supraorbital level and posteriorly at the level of the external occipital protuberance.

Once placed on the MR scanner bed, the patient is monitored for vital signs and provided with an alarm bell in case of emergency. The transducer helmet is then filled with water and the bed is positioned inside the gantry.

The preparation time goes from the patient arrival to the beginning of MRI acquisitions (localizer).

### Pre-treatment planning time

The treatment begins with the acquisition of an MRI volumetric sequence (T1 3D IR FSPGR, BRAVO) with multiplanar reconstructions. The MR images acquired with the CT images previously obtained for the measurement of the skull density ratio (SDR) between the cortical bone and the cancellous bone are then merged. The fusion of the images allows to identify intracranial calcifications and areas containing air that can block the transmission of ultrasounds and are marked as “no-pass zone” for the ultrasound beams. The system then calculates the number of active transducer elements for the treatment (acceptable if ≥ 700) and the actual surface (acceptable if ≥ 250cm^2^). In addition, fiducial markers are placed on the 3 planes to identify any head movement during treatment.

The initial coordinates for the Vim are positioned according to the canonical stereotactic coordinates (indirect targeting).

The planning time goes from the acquisition of MRI volumetric sequence to the first alignment sonication (excluded).

### Treatment time (sonications)

By sonication, we mean each administration of a beam of waves designed to reach the designated target. The sonication procedure includes 3 steps.

The first (alignment) includes short sonications at very low energy and temperature (up to 45 °C) to confirm that the sonication point coincides with the target coordinates. The second step (confirmation) includes sonications with increasing parameters of energy and power to reach temperatures necessary to obtain a neuromodulation effect (stupor) and confirm the effectiveness of the treatment in the target set and the possible presence of adverse effects. Based on the clinical response, the target is repositioned if necessary, considering the somatotopic distribution of the Vim neurons and nearby structures (internal capsule, ventrocaudal nucleus, etc.). In this stage, temperatures of about 46–50° C are reached. The number of movements is determined by the sum of the movements of the target coordinates carried out until an adequate therapeutic result is reached, which consists of the disappearance of the tremor in the absence of side effects.

In the last step (treatment)the energy, the sonication time, and the number of sonications are increased and modulated to reach maximum temperatures of 60° C to obtain coagulation necrosis (ablation) at the target level. Generally, the lesion is effective with at least 2 sonications that have reached a temperature > 56° C. Subsequently, a targeted T2 acquisition is made at the ablation site for control.

At the end of the treatment, after the water has been drained from the helmet and the stereotactic frame has been removed, the patient can get up and undergo a final complete neurological examination before being transferred back to the ward for observation until the next day.

The average duration of treatment, from positioning to the end of the treatment, is approximately 3 h.

The treatment time goes from the first alignment sonication to the last sonication.

#### Statistical analysis

Data were collected, organized, and analyzed through XLSTAT 2017: Data Analysis and Statistical Solution for Microsoft Excel (Addinsoft, Paris, France 2017). Descriptive statistics were calculated for all variables. All numerical data were expressed as mean ± standard error of the mean. The Student’s *t*-test was used to determine group differences between variables, and the Wilcoxon test was used if the assumptions of normality for the t-tests were not fulfilled. The assumption of normality was evaluated using the Shapiro–Wilk and Kolmogorov–Smirnov tests. The Chi-square test was used to compare the distribution of categorical variables. A multiple comparisons with repeated measures test was used to asses variance of continuous variables. Statistical significance was set with a *p* values ≤ 0.05.

## Results

In Group A (9 females, 21 males), 15 patients had TE while 15 PD, the mean age was 66.5 years (C.I. 95%: 62.91–70.09) and the mean SDR value was 0.44 (C.I. 95%: 0.41–0.47). In Group B (7 females, 23 males), in which 12 patients had TE while 18 PD, the mean age was 71 years (C.I. 95%: 68.01–74.06) and the mean SDR value was 0.43 (C.I. 95% 0.41–0.44). In Group C (6 females, 24 males), in which 11 patients had TE while 19 PD, the mean age was 67.1 years (C.I. 95%: 62.93–71.31) and the mean SDR value was 0.41 (95% C.I.: 0.38–0.44). Findings are summarized in Table 1.

For Group A, the preparation time was 120.6 min (95% C.I.: 108.6–132.5), the pre-treatment planning time was 51.4 min (95% C.I.: 45.7–57.1), and the treatment time was 105.2 min (95% C.I.: 90.3–120.1). The time required for displacements was 3.1 min (95% C.I.: 1.8–4.4) and for sonications was 14.1 min (95% C.I.: 12.4–15.9). For Group B, the preparation time was 105.5 min (95% C.I.: 92.7–118.2), the pre-treatment planning time was 46.2 min (95% C.I.: 40.7–51.7), and the treatment time was 89.5 min (95% C.I.: 75.6–103.5). The time required for displacements was 3.0 min (95% C.I.: 1.8–4.0) and for sonications was 13.2 min (95% C.I.: 11.6–14.7). For Group C, the preparation time was 101.9 min (95% C.I.: 90–113.8), the pre-treatment planning time was 52.3 min (95% C.I.: 45.7–58.9), and the treatment time was 71.7 min (95% C.I.: 60.9–82.5). The time required for displacements was 1.7 min (95% C.I.: 0.8–2.6) and for sonications was 10.6 min (95% C.I.: 9.3–11.9). Table 2 provides the summary statistics for the required time for each phase of the MRgFUS Thalamotomy for each group.

**Table 2:** The time required for each phase of the treatment for each group

Groups	*A*	*B*	*C*	*P-*value
SDR	0.44	0.43	0.41	1 A–B 0.6 A–C 1 B–C
Preparation time (min)	120.6	105.5	101.9	0.79 A–B 0.73 A–C 0.04* B–**C**
Planning time (min)	51.4	46.2	52.3	0.30 A–B 1.00 A–C 0.19 B–C
Treatment time (min)	105.2	89.5	71.7	0.57 A–B 0.01* A–**C** 0.17 B–C
Target Displacements (n)	3.1	3.0	1.7	1.00 A–B 0.18 A–C 0.29 B–C
Sonications (n)	14.1	13.2	10.6	1.00 A–B 0.01* A–C 0.04* B–**C**

*SDR*, skull density ratio

^*^Indicates *P* < 0.05

In all groups the FTM scores significantly improved immediately after treatment (Group A: from 5.4 to 0.6; Group B 5.3 to 0.5; Group C 5.6 to 1.3) and the improvement remained substantially stable at the following 1-month, 6-month, and 1-year follow-up. The trends of the FTM scores during follow-up is shown in Table 3.

**Table 3:** FTM tremor score (part A of the arm treated) during each follow-up (mean values)

Groups	*A*	*B*	***C***
Baseline	5.4	5.3	5.6
24 h	0.6^*^	0.5^*^	1.3^*^
1 month	1.2^*^	0.8^*^	1.3^*^
6 month	1.4^*^	1.2^*^	1.8^*^
1 year	1.5^*^	1.7^*^	1.9^*^

^*^Indicates *P* < 0.05 compared with the baseline

Patient-related complications were evaluated by days of hospitalization, the number of complications, and the number of relapses. The mean number of days of hospitalization was 3.8 (95% C.I.: 3.4–4.3) for Group A, 3.2 (95% C.I.: 3.1–3.4) for Group B, and 3.5 (95% C.I.: 3.1–3.8) for Group C. Thalamotomy related complications were experienced by 9 patients in Group A, 2 in Group B, and 5 in Group C. Lastly, tremor relapse was found in 7 patients in Group A, 3 in Group B, and 2 in Group C. Patient-related complications are exposed in Table 4.

**Table 4:** Patient-related complications

Group	A	B	C	*P*-value
Days of hospitalization	3.8	3.2	3.5	0.04* A–B 0.73 A–C 0.79 B–C
Complications (np)	9	2	5	0.04* A–**B** 0.36 A–C 0.42 B–C
Tremor relapse (np)	7	3	2	0.29 A–B 0.14 A–C 1.00 B–C

*np*, number of patients

^*^Indicates *P* < 0.05

## Discussion and conclusions

In our study, we reported the results of our experience in the MRgFUS treatment of refractory tremor in 90 patients (38 ET, 52 PD) and how the clinical and procedural parameters considered have improved in the light of the operator’s greater experience with the technique.

For a very long time, neurological movement disorders have been treated with radiofrequency ablation, deep brain stimulation (DBS), or stereotactic radiosurgery (Gamma-Knife). Nevertheless, these techniques are burdened with various side effects and surgical risks [22]. Focused ultrasound thalamotomy is an innovative technique for the treatment of neurological diseases such as essential tremor and Parkinson’s disease refractory to drug therapy and it is characterized by fewer procedural complications [2–6, 10–16, 18, 19, 21].

Several studies in the literature, especially in the surgical field, evaluate the operator’s experience as a fundamental parameter for complications and, more generally speaking, for the outcome [10, 14, 17].

In our study, a total of ninety patients were examined, divided into three homogeneous groups by pathology, sex, and age. The first group included patients enrolled from February 2018 to January 2019, the second group from January 2019 to September 2019, and the third from September 2019 to July 2020.

**Fig. 1:**
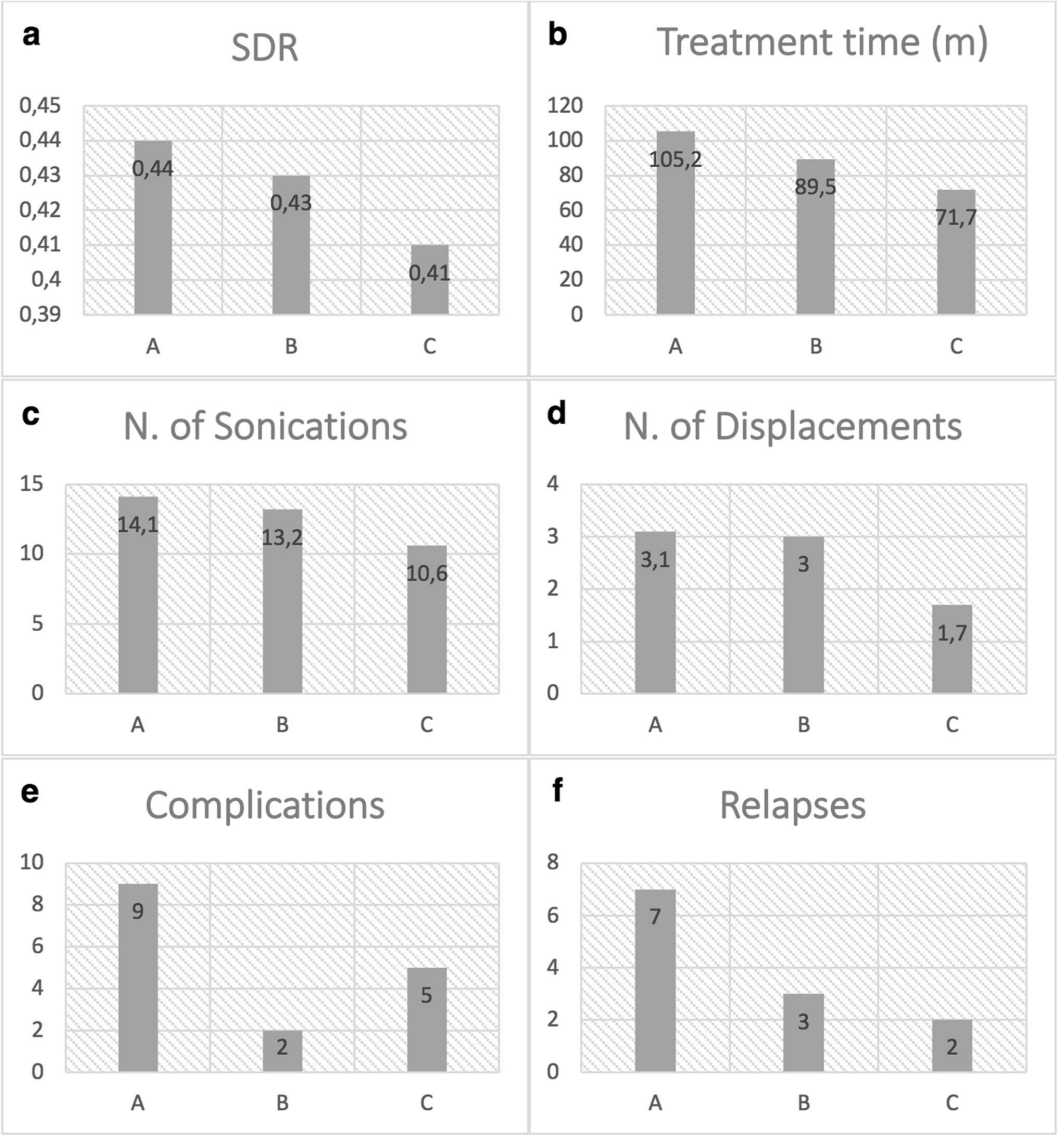
Histograms showing the mean SDR value (**a**), the treatment time in minutes (**b**), the number of sonications (**c**), and the number of displacements (**d**). The number of patients who experienced a least one side effect and who had the relapse of the tremor are reported in the histograms in **e** and **f**, respectively

For each group, we evaluated different procedural parameters and how the operator’s experience has affected them over this period. The skull density ratio (SDR) between cortical bone and cancellous bone (values > 0.3 are considered suitable for treatment) shows a decrease from 0.44 in Group A to 0.41 in Group C (Fig. 1a). Over time, we have expanded the selection of patients with gradually lower SDR, without repercussions on complications and procedural times. This was possible thanks to the operator’s greater competence and management of the method.

In the three groups, there was a marked decrease in the time of preparation, planning, and treatment from Group A to Group C. Regarding the time of patient preparation, we started with Group A with an average time of 120.6 min, Group B with 105.5 min to arrive at Group C with 101.9. During the planning time, we started with Group A with an average of 51.4 min, and Group B with 46.2 min to arrive at Group C with 52.3. The most significant reduction in time was highlighted during the treatment, passing from Group A with 105.2 to Group B with 89.5 to Group C with 71.7 (Fig. 1b). The application of this method has led the operator to become more familiar not only with the imaging guide but also with some parameters strictly related to ultrasound management such as energy, power, and sonication time. Time optimization is essential since the patient is awake during the procedure and may request, in any circumstance, to suspend it all. At the start of the trial, some patients asked for treatment to be discontinued. The number of sonications (Fig. 1c) and displacements (Fig. 1d) also follows the trend of the previous parameters: Group A had displacements equal to 3.1, Group B to 3.0, and Group C to 1.7. Regarding the number of sonications instead, Group A had 14.1, Group B 13.2, and Group C 10.6. This sharp decrease can be explained with a change in the treatment strategy: initially, the movements were more cautious and of a small entity (about 0.3 mm), with the experience we moved less and, if necessary, the displacements were greater (about 0.5–1 mm) thanks to the greater confidence with the thermometry evaluation, setting of sonication parameters, and thalamic functional anatomy..

Lastly, while the days of hospitalization remained constant in the three groups (Group A 3.8, Group B 3.2, and Group C 3.5), we observed a net decrease in the incidence of complications (Fig. 1e) and relapses (Fig. 1f). In Group A, 9 patients developed complications, 2 in Group B, and 5 in Group C; while 7 patients relapsed in Group A, 3 in Group B, and 2 in Group C.

In other studies, they also evaluated the plateau or cut-off of cases beyond which surgical performance becomes stable. Regarding thalamotomy using MRgFUS, although there is a reduction in procedural parameters, there are variables related to the patient that ensure that this plateau is not reached.

As a secondary objective of our study, in addition to evaluating the impact of operator experience on procedural management, we also evaluated possible differences in the clinical outcome.

In fact, the efficacy of the VIM ablation procedure with MRgFUs has been amply proven by numerous studies and in particular our experience has been previously published too [7, 8]. Our clinical results in this study confirm an immediate reduction in FTM scores with improvement stability around 70%. Not many studies report long-term results but the recent report by Cossgrove et al. [9] documents an improvement in tremor at 60 months of 73% for the part A of FTM and 40% for parts A and B. The trend of a minimum increase in scores compared to the immediate post-procedural evaluation is due often to occurrence of recurrences which, in literature, amount to between 8 and 11%. All the factors determining the onset of recurrences are still not completely clear, even if it is believed that the procedural optimization of the target could be an important element.

## Conclusions

This study set out to determine whether or not the operator experience may affect the clinical outcome of the MRgFUS Thalamotomy performed by neuroradiologists, since it embraces different aspects of radiology and surgery. The results of our investigation demonstrate how experienced and trained physicians may achieve more rapid and effective results after an initial time frame. Interestingly, not only the procedure time, but also the procedure-related complications and the relapse rate sensibly diminish after 20 months.

However, our study has some limitations to report, including the lack of stratification by age and by pathology and duration of the disease.

Further work needs to be done, in collaboration with other centers, to establish a defined learning curve and hopefully, a learning pathway, for this novel technique.

## Data Availability

The data that support the findings of this study are available from the corresponding author (LP), upon reasonable request.

## References

[1] Abe K, Horisawa S, Yamaguchi T (2021). Focused ultrasound thalamotomy for refractory essential tremor: a Japanese multicenter single-arm study. Neurosurgery.

[2] Agrawal M, Garg K, Samala R (2021). Outcome and complications of MR guided focused ultrasound for essential tremor: a systematic review and meta-analysis. Front Neurol.

[3] Arrigoni F, Barile A, Zugaro L (2017). Intra-articular benign bone lesions treated with Magnetic Resonance-guided Focused Ultrasound (MRgFUS): imaging follow-up and clinical results. Med Oncol.

[4] Arrigoni F, Napoli A, Bazzocchi A (2019). Magnetic-resonance-guided focused ultrasound treatment of non-spinal osteoid osteoma in children: multicentre experience. Pediatr Radiol.

[5] Barile A, Arrigoni F, Zugaro L (2017). Minimally invasive treatments of painful bone lesions: state of the art. Med Oncol.

[6] Bruno F, Catalucci A, Arrigoni F (2020). An experience-based review of HIFU in functional interventional neuroradiology: transcranial MRgFUS thalamotomy for treatment of tremor. Radiol Med.

[7] Bruno F, Catalucci A, Arrigoni F, Sucapane P, Cerone D, Cerrone P, Ricci A, Marini C, Masciocchi C (2020). An experience-based review of HIFU in functional interventional neuroradiology: transcranial MRgFUS thalamotomy for treatment of tremor. Radiol Med.

[8] Bruno F, Catalucci A, Varrassi M, Arrigoni F, Gagliardi A, Sucapane P, Cerone D, Pistoia F, Ricci A, Marini C, Masciocchi C (2020). Bilateral MRgFUS thalamotomy for tremor: a safe solution? Case report and review of current insights. Clin Neurol Neurosurg.

[9] Cosgrove, G. R., Lipsman, N., Lozano, A. M., Chang, J. W., Halpern, C., Ghanouni, P., Eisenberg, H., Fishman, P., Taira, T., Schwartz, M. L., McDannold, N., Hayes, M., Ro, S., Shah, B., Gwinn, R., Santini, V. E., Hynynen, K., & Elias, W. J. (2022). Magnetic resonance imaging–guided focused ultrasound thalamotomy for essential tremor: 5-year follow-up results, *Journal of Neurosurgery* (published online ahead of print 2022). 10.3171/2022.6.JNS212483 PMC10193464 35932269

[10] Daube P, Cagnazzo F, Barreau X (2021). Influence of operator experience on the technical and clinical results of Woven EndoBridge endovascular treatment for intracranial aneurysms. Clin Neurol Neurosurg.

[11] Geraedts VJ, van Ham RAP, Van Hilten JJ (2021). Intraoperative vs. postoperative side-effects-thresholds during pallidal and thalamic DBS. Front Neurol.

[12] Jameel A, Bain P, Nandi D (2021). Device profile of exAblate Neuro 4000, the leading system for brain magnetic resonance guided focused ultrasound technology: an overview of its safety and efficacy in the treatment of medically refractory essential tremor. Expert Rev Med Devices.

[13] Kim MJ, Park SH, Chang KW (2021). Technical and operative factors affecting magnetic resonance imaging-guided focused ultrasound thalamotomy for essential tremor: experience from 250 treatments. J Neurosurg.

[14] Lin YY, Chen RS, Huang YZ (2022). Impact of operator experience on transcranial magnetic stimulation. Clin Neurophysiol Pract.

[15] Masciocchi C, Arrigoni F, Ferrari F (2017). Uterine fibroid therapy using interventional radiology mini-invasive treatments: current perspective. Med Oncol.

[16] Masciocchi C, Conchiglia A, Gregori LM (2014). Critical role of HIFU in musculoskeletal interventions. Radiol Med.

[17] Nagakawa Y, Nakamura Y, Honda G (2018). Learning curve and surgical factors influencing the surgical outcomes during the initial experience with laparoscopic pancreaticoduodenectomy. J Hepatobiliary Pancreat Sci.

[18] Ondo WG (2020). Current and emerging treatments of essential tremor. Neurol Clin.

[19] Pineda-Pardo JA, Urso D, Martinez-Fernandez R (2020). Transcranial magnetic resonance-guided focused ultrasound thalamotomy in essential tremor: a comprehensive lesion characterization. Neurosurgery.

[20] Sharma S, Pandey S (2020). Treatment of essential tremor: current status. Postgrad Med J.

[21] Valentino F, Cosentino G, Maugeri R (2020). Is transcranial magnetic resonance imaging-guided focused ultrasound a repeatable treatment option? Case report of a retreated patient with tremor combined with parkinsonism. Oper Neurosurg (Hagerstown).

[22] Wang KL, Ren Q, Chiu S (2020). Deep brain stimulation and other surgical modalities for the management of essential tremor. Expert Rev Med Devices.

